# Chewing Gum Versus Standard Care for Enhanced Bowel Recovery After Cesarean Section: A Randomized Clinical Trial

**DOI:** 10.7759/cureus.68210

**Published:** 2024-08-30

**Authors:** Brundha N, Aruna Biradar, Neelamma Patil, Rajasri G Yaliwal, Priyanka Biradar, Namita Gupta, Sharanabasava S Kulkarni

**Affiliations:** 1 Obstetrics and Gynaecology, Shri B. M. Patil Medical College and Research Centre, Vijayapura, IND

**Keywords:** sham feeding, early gut motility, post operative care, caesarean section, gum chewing

## Abstract

Background and objective

Cesarean sections (CS) are common and occasionally critical surgical procedures. Nausea, vomiting, and postoperative ileus (POI) frequently occur in patients undergoing cesarean delivery with regional anesthesia. These issues affect patient comfort, slow down wound healing, and prolong hospital stays. Studies have employed various strategies to address these challenges. Chewing gum post-surgery is a cost-effective approach to stimulate intestinal movement, promoting early initiation of oral intake, early mobilization, shorter hospital stays, and reduced overall hospitalization time. In this study, we aimed to investigate the potential benefits of chewing gum in this patient population.

Methodology

We randomly assigned a total of 314 women scheduled for elective or emergency lower segment CS (LSCS) to either a gum-chewing group (Group A, n=157) or a control group receiving standard postoperative care (Group B, n=157). Participants in the gum-chewing group chewed sugar-free gum for one hour postoperatively in sessions lasting 15 minutes each, continuing until bowel sounds resumed, and were allowed to have oral sips of water. In contrast, the control group's bowel sounds were checked every half hour till they appeared, and women were allowed to have sips of water once the bowel sounds were heard. The primary comparisons between the groups focused on the timing of first bowel sounds, first passage of flatus, and first bowel movement. Secondary endpoints included time to mobilization, removal of the catheter, and subjective sense of well-being.

Results

The average age of women in both groups was 24 years. There were no statistically significant differences between the study and control groups concerning age, parity, occupation, type of CS, reasons for CS, skin incision, or intraperitoneal adhesions. In Group A, the mean time for bowel sounds to appear was 3.39 hours, compared to 6.91 hours in Group B. For flatus passage, the mean time was 12.74 hours in Group A and 20.51 hours in Group B. Stool passage took an average of 41.59 hours in Group A and 64.03 hours in Group B.

Conclusions

Chewing gum, a type of simulated eating, is linked to faster restoration of gastrointestinal function following CS. This study elucidates the mechanisms behind the benefits of chewing gum and explores its potential in diverse surgical populations. Ultimately, integrating chewing gum into postoperative care may offer a valuable tool for enhancing patient outcomes and accelerating recovery, provided it is used in conjunction with comprehensive and personalized postoperative care strategies. It is a cost-effective approach that accelerates the recovery of intestinal movement post-surgery, decreasing the length of hospital stay and overall burden on patients.

## Introduction

Childbirth is the most stressful, exhilarating, and fulfilling moment in a mother’s life. Every labor experience is different and deserves to be celebrated. The delivery methods include both vaginal birth and cesarean sections (CS). CS can be planned or performed in an emergency. In India, there is an alarming trend of growth in the number of CS, and concerns are being raised. The proportion of cesarean deliveries has dramatically increased to 17% (2015-16) and 21.5% (2019-21) from just 3% in 1992-93 [1]. CS is generally associated with increased maternal surgical risks for the current and subsequent pregnancies compared to spontaneous vaginal delivery. Recovery following CS can present challenges in the restoration of gut motility.

Generally, following abdominal surgery, small intestine activity returns to normal function in a few hours, gastric activity returns in 24-48 hours, and colon activity returns in 48-72 hours [2]. Some degree of adynamic ileus follows every laparotomy, which also holds true for cesarean deliveries. Following abdominal surgery, postoperative ileus (POI) is a common occurrence that results in a temporary cessation of bowel motility. It can cause extended hospital stays, delay recovery, and increase healthcare costs. The overall incidence of POI for all operative procedures in the abdomen is 9.2% [3].

The surgical goal for preventing POI involves striving to minimize bowel manipulation, avoiding excess IV fluids or profound hypovolemia, and limiting the duration of surgery. Chewing gum has recently been recognized as a novel, convenient, easily accessible, cost-effective method to reduce POI. Chewing gum releases gastrointestinal hormones responsible for bowel motility and stimulates intestinal movement through the cephalic-vagal reflex. This leads to the early restoration of bowel sounds, flatus passage, and appetite return [4].

We conducted this study to evaluate the efficacy of chewing gum in promoting bowel mobility following cesarean delivery. The study seeks to contribute valuable insights in terms of optimizing postoperative care protocols for women undergoing CS by investigating the potential benefits of chewing gum in this specific patient population. The findings may influence clinical practice guidelines, offering healthcare providers a simple and cost-effective intervention to improve patient outcomes and overall satisfaction during post-cesarean recovery.

## Materials and methods

The study included women meeting specific criteria - maternal age between 18 and 40 years, either primigravida or multigravida, at a gestational age of more than 28 weeks - who agreed to give written and informed consent and scheduled for emergency or elective CS under spinal anesthesia between 8:00 AM and 6:00 PM at Shri B.M. Patil Medical College and Research Hospital, Vijayapura, Karnataka. The study was conducted from April 2023 to October 2024. Ethical clearance was obtained (IEC/762/2022-23) and the study is registered in the clinical trials registry - India (CTRI/2023/03/051199). These criteria aimed to ensure a consistent patient profile for evaluating procedure and postoperative care outcomes.

The study excluded participants with high-risk pregnancies (e.g., pregnancy-induced hypertension, diabetes during pregnancy), history of gastrointestinal surgeries or chronic renal disease, electrolyte imbalances, previous CS under general anesthesia or subsequent laparotomies, intraoperative complications (e.g., postpartum hemorrhage, bladder or bowel injuries, bowel adhesions), operations exceeding two hours, and conditions requiring prolonged nil by mouth. A thorough history and general physical examination were conducted. The duration of the surgery and any significant intraoperative findings were recorded. Preoperative electrolytes were performed to omit electrolyte disturbance (Na, K, Ca, M).

Patient groups

**Figure 1:**
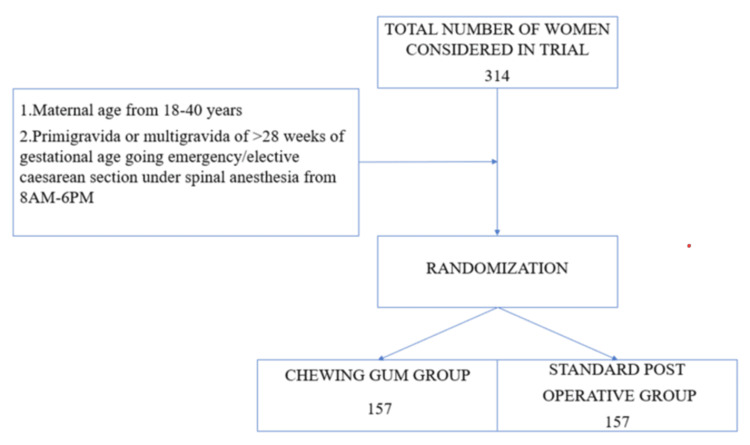
Flow chart showing the randomization of patients

A total of 314 women were randomized into the study group (157) and control group (157) by using the computer-generated randomized program as shown in Figure 1.

Group A

**Figure 2:**
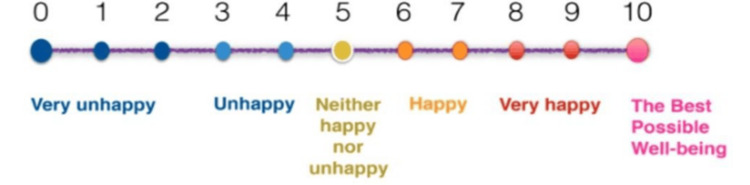
Visual analog scale

Participants in the study group study began gum chewing within one hour after surgery upon transfer to the postoperative ward. They had sugar-free gum with a mixed fruit flavor for 15-30 minutes every two hours until bowel sounds resumed. The timing of the first flatus and stool passage was self-reported by the patients. Gum chewing was restricted for Group A from 10:00 PM to 8:00 AM. A visual analog scale assessed their sense of well-being at the time of discharge. Mothers who participated in the study were shown the visual analog scale and were asked to grade their general well-being post-surgery considering the pain, hydration, diet, mobility, and emotional well-being (Figure 2).

Group B

According to the standard postoperative care protocol, bowel sounds are checked every 30 minutes in the recovery after one hour of the cesarean section. Once the bowel sounds are heard, the patients are allowed to have sips of water. The time of appearance of bowel sounds is recorded. Next, the time of appearance of the first flatus and stool passage is noted after allowing the patient to have water. A visual analog scale assessed their sense of well-being at the time of discharge (Figure 2).

Statistical analysis

The collected data were recorded in a Microsoft Excel spreadsheet and analyzed using the SPSS Statistics Version 20 (IBM Corp., Armonk, NY). Results were reported as mean ± standard deviation (SD), counts, percentages, and graphical representations. For comparing normally distributed continuous variables in groups, the independent t-test was applied; for non-normally distributed variables, the Mann-Whitney U test was used. Categorical variables between groups were compared using the chi-square test. A p-value less than 0.05 indicated statistical significance, and all analyses were conducted accordingly.

## Results

Most of the women were in the age group of 20-24 years: 78 (49.70%) in Group A and 73 (46.50%) in Group B, with an age range of 18-40 years in both groups (in Group A, the average age of women was 24.52 3.93 years, while in Group B, it was 24.59 4.15 years; p=0.6). Most of the women attending our institution for delivery were from rural areas, i.e., 82 (52%) from Group A and 84 (54%) from Group B (p=0.863). Among 314 women, 208 were multigravida, and 106 were primigravida, with no statistically significant difference between both groups (p=0.34). The mean gestational age was 38.92 ±1.43 in Group A and 39.01 ±1.26 in Group B (p=0.588).

The total number of emergency lower segment CS (LSCS) performed was 37 (87%) in Group A and 25 (80%) in Group B. The number of elective LSCS was 52, out of which 20 (13%) were in Group A and 32 (20%) were in Group B (p 0.06). Of these CS, the most common indication was noted to be previous LSCS: a total of 68 (43%) and 71 (45%) in the study and control groups, respectively. The second most common indication was fetal distress: 25 (16%) and 24 (15%) women in the study and control groups, respectively. As per inclusion criteria, all women were given spinal anesthesia in both groups, and a Pfannenstiel incision was used in both sets. Twenty (13%) women in the study group and 18 (11.5%) in the control group had adhesions (p=0.696).

The total number of gums chewed by the participants in Group A was 157: 32 (20.38%) women used only one chewing gum before the onset of bowel movements, flatus, or feces; 104 (66.24%) required two chewing gums; 19 (12.10%) required three chewing gums; and only one woman each required four and five chewing gums, respectively. Out of 47 women with one previous LSCS, 38 (80%) required two chewing gums. Among 19 women with two previous LSCS, seven (36.84%) required two chewing gums, and 11 (57.89%) required three.

Table 1 shows the mean duration of the time taken for the appearance of bowel sound, first flatus, and first stools following the surgery, as well as the time of oral intake, time taken for mobilization, and catheter removal.

**Table 1:** Mean duration of primary and secondary parameters in Group A and Group B *Statistically significant

	Group	N	Mean	Std. deviation	P-value
Time taken for first bowel sound, hours	Group A	157	3.395	1.252	0.0001*
	Group B	157	6.911	1.1976	
Time taken for first flatus, hours	Group A	157	12.74	4.841	0.0001*
	Group B	157	20.51	5.192	
Time taken for first stools, hours	Group A	157	41.59	12.351	0.0001*
	Group B	157	64.03	33.413	
Time of oral intake following surgery, hours	Group A	157	3.459	1.1698	0.0001*
	Group B	157	6.949	1.2419	
Time of mobilization, hours	Group A	157	9.35	3.398	0.0001*
	Group B	157	12.95	3.415	
Time of catheter removal, hours	Group A	157	27.17	11.758	0.0001*
	Group B	157	42.05	12.92	
Sense of well-being (graded as per the visual analog scale)	Group A	157	9.69	0.619	0.0001*
	Group B	157	7.18	0.732	
Duration of hospital stay, days	Group A	157	4.82	1.461	0.0001*
	Group B	157	6.4	1.683	

**Figure 3:**
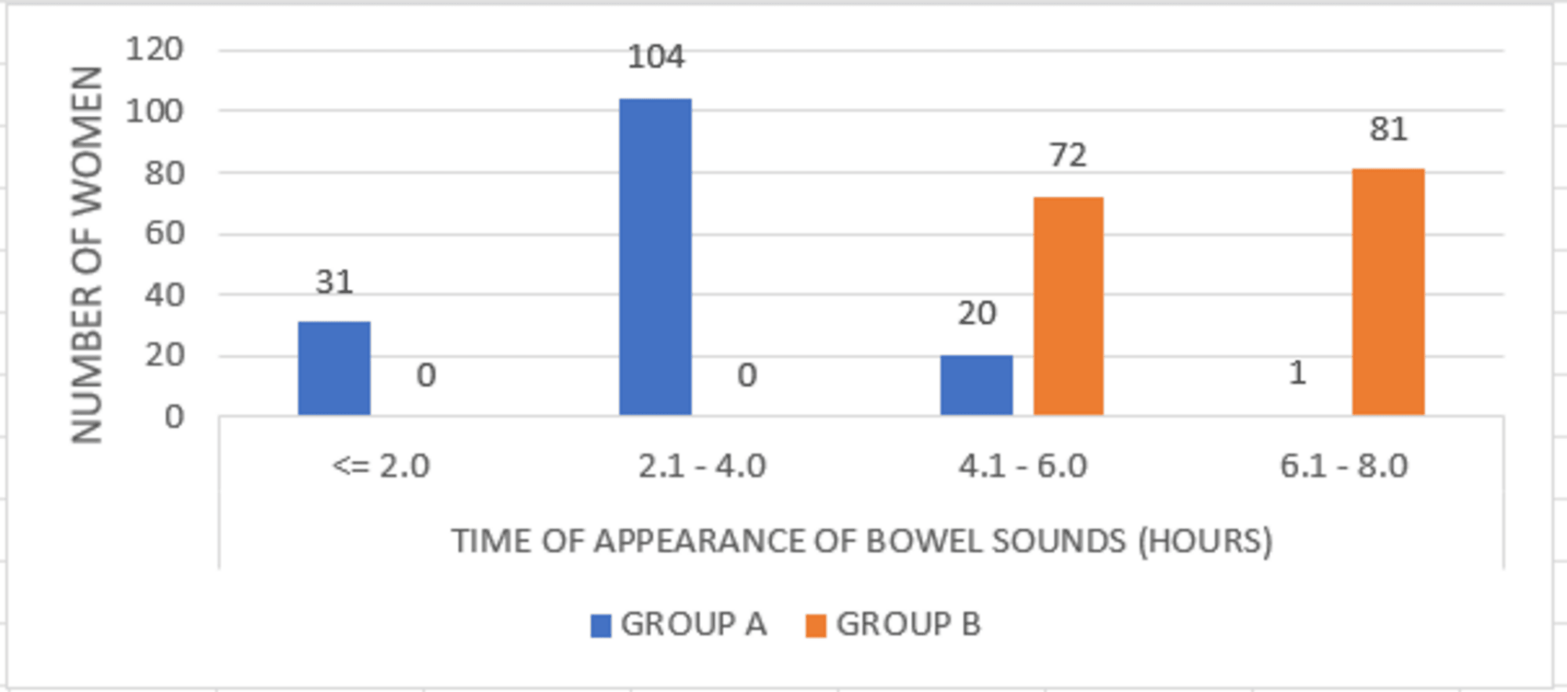
Comparison of the time of appearance of bowel sounds between Group A and Group B P=0.0001 (Pearson's chi-square test)

The time taken for the bowel sounds to appear was less than two hours for 31 (19.70%) women in the case group. At the same time, in the majority of the women (66.20%) in the case group, bowel sounds appeared at two to four hours. In the control group, bowel sounds appeared in four to six hours in 72 women (45.90%), while they appeared in six to eight hours in 81 (51.60%) women. The average time taken for the occurrence of bowel sounds among the case and control was 3.39 hours and 6.91 hours, respectively, and the difference was statistically significant (p=0.0001), as shown in Figure 3.

**Figure 4:**
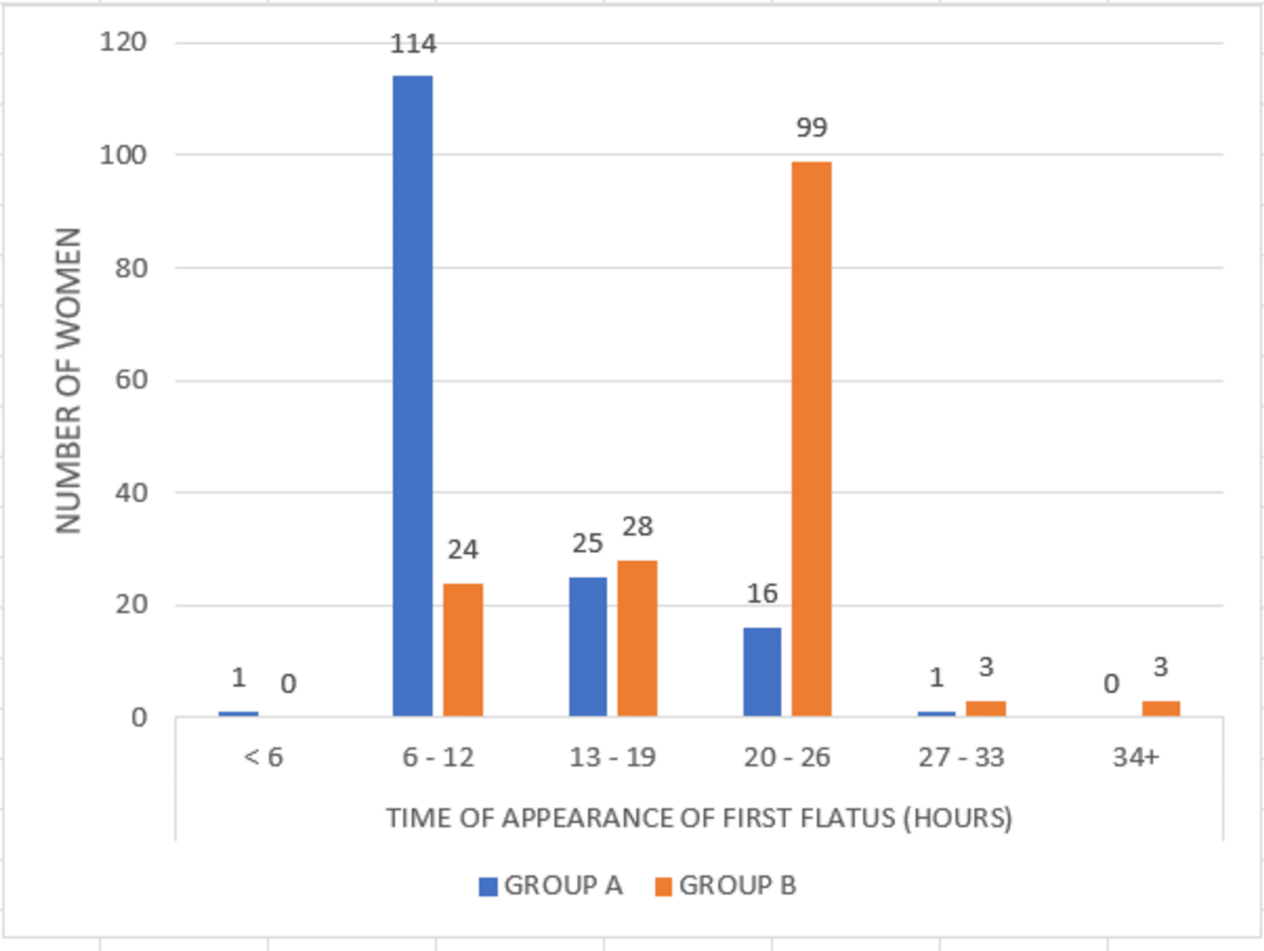
Comparison of the time of appearance of the first flatus between Group A and Group B P=0.0001 (Pearson's chi-square test)

In the case group, the first flatus occurred in 6-12 hours following surgery in the majority of the women, while it occurred in 20-26 hours in the control group. The mean time taken for the appearance of the first flatus among case and control were 12.74 hours and 20.51 hours, respectively, with a significant difference (p=0.0001), as shown in Figure 4.

**Figure 5:**
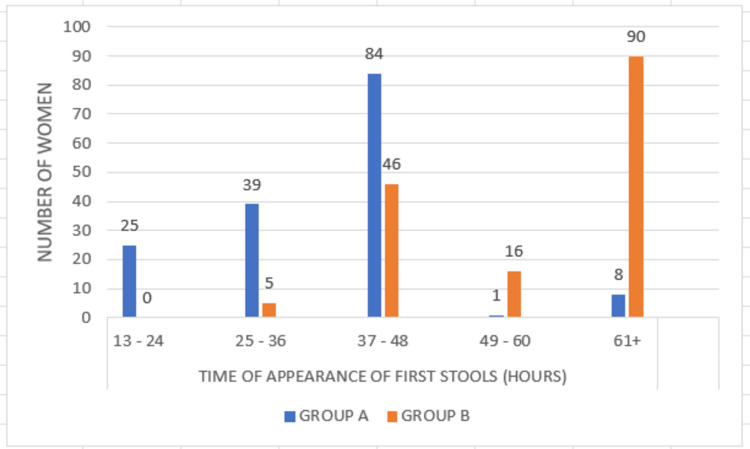
Comparison of the time taken for first stools between Group A and Group B P=0.0001 (Pearson's chi-square test)

In the case group, the time taken for stools following surgery was 37-48 hours in the majority of women, while it was 60+ hours in the control group. The mean time taken for stools following surgery among the case and control were 41.59 hours and 64.03 hours, respectively, with a significant difference (p=0.0001), as shown in Figure 5.

Following surgery, complications such as abdominal distension were noted in one woman in the case group and six women in the control group, while nausea and vomiting were seen in three women in the case and 13 women in the control group (p=0.12). The visual analog scale scores showed that women chewing gum had a better sense of well-being than those in Group B. The mean duration of hospital stays was 4.82 days in the study group vs. 6.4 days in the control group. Group A's hospital stay was substantially shorter than Group B's.

## Discussion

Our study involved 314 pregnant women undergoing LSCS who met the inclusion criteria at Shri B.M. Patil's Medical College, Hospital and Research Centre. Among them, 157 were included in the gum-chewing group, and 157 were placed in the standard postoperative group. The study's findings, which were consistent with those of other studies published in the literature, showed that there was no statistically significant difference between the two groups regarding their general characteristics, age groups, parity, occupation, LSCS/prior abdominal surgery, type of cesarean section, indication of cesarean section, and type of anesthesia.

By stimulating intestinal myoelectric activity and activating cephalic vagal pathways, chewing gum improves the recovery of bowel motility through physiological mechanisms that block the activation of µ opioid receptors in the gastrointestinal tract. Chewing mimics eating, which increases electrical signals in the intestines and promotes muscle contractions, aiding in the movement of contents. It also activates neural pathways involving the vagus nerve, sending signals from the brain to the gut to enhance digestive activity. This process releases digestive hormones and neurotransmitters that inhibit µ opioid receptors, preventing a slowdown in bowel movements and thereby improving bowel motility. A study by Elkan Kiyat and Kahyaoglu Sut investigating how post-CS xylitol gum chewing affects bowel movements showed positive results. They concluded that chewing gum containing xylitol in the early postpartum period is an effective and convenient way to induce bowel movements sooner [5].

In our study, there was no statistical significance between both groups regarding the age groups of the participants. Similar results were noted in other studies. In a prospective randomized controlled trial by Manisha and Duhan, the mean age in Group A women was 24.86 ±3.89 years while it was 25.28 ±3.34 years in Group B [6]. Similarly, in a randomized study by Mutlag et al. from Egypt, the mean age of Group A women was 23.62 ±3.55 years while it was 22.82 ±4.31 years in Group B [7]. Also, a study done by Ajuzieogu et al. showed that the mean age in Group A women was 25.0 ±6.4 years and 25.5 ±6.0 years in Group B [8].

The time of appearance of bowel sounds depends on the duration of the procedure, the impact of anesthesia, the handling of the gut, the presence of intraperitoneal adhesions during the procedure, and blood loss during surgery, as well as any prior abdominal or CS history [9]. In this study, the time for the first appearance of bowel sounds in the study group was 3.39 hours, while it was 6.91 hours in the control group. This difference was statistically significant (p=0.0001). These findings align with other studies, such as a randomized control trial by Manisha and Duhan, in which the mean time taken for the appearance of bowel sounds was 3.27 hours in the gum-chewing group and 8.22 hours in the control group [6]. Similarly, in a study by Mutlag et al., the mean time for the appearance of bowel sounds was 11.8 hours in the gum-chewing group and 16.96 hours in the control group [7]. Also, in the study by Ajuzieogu et al. from Nigeria, the mean time taken for bowel sounds was relatively higher: 21.9 hours and 26.1 hours in the gum-chewing and control groups, respectively [8].

After surgery, the return of motility was typically first observed in the small bowel in less than 24 hours, then in the stomach between 24 and 48 hours, and finally in the large bowel after more than 48 hours [10-15]. The study group experienced the first flatus passage at 12.74 hours, whereas the control group experienced it at 20.51 hours. These findings are comparable to those observed in other studies. In a randomized control trial by Manisha and Duhan, the mean time taken for the appearance of flatus was 9.77 hours in the chewing gum group and 17.15 hours in the standard postoperative group. Similarly, in the study by Mutlag et al., the mean time for the appearance of flatus following surgery was 13 hours in Group A and 27.55 hours in Group B. Also, in the study by Ajuzieogu et al., the mean time taken for flatus to appear was higher. 24.8 hours for the gum-chewing group and 30.0 hours for the control group.

Similar to previous research in the literature, the study group's first bowel movement following surgery occurred 41.59 hours afterward, while the control group's first bowel movement occurred 64.03 hours later. Notably, one randomized controlled trial also assessed the effects of chewing xylitol-containing and xylitol-free gum on gastrointestinal recovery following cesarean section [11]. The results suggested that xylitol-containing gum might be preferable to xylitol-free gum. More research is warranted in this area. The sweetening ingredients in sugar-free gum, such as sorbitol and xylitol, may promote bowel movement and have a laxative effect. However, there is conflicting evidence regarding how these sugar substitutes in sugar-free gum affect bowel function [12].

Study strengths

This study explores an unconventional yet simple method: chewing gum to potentially enhance postoperative recovery, offering a novel addition to existing care protocols. Chewing gum is an inexpensive intervention, making it a cost-effective option. This study addresses a practical aspect of postoperative care, with the potential benefits including reduced discomfort and quicker return of bowel function, which may enhance overall patient satisfaction.

Study limitations

Our sample size was relatively small and hence we recommend larger studies to gain deeper insights into the topic. Differences in postoperative care practices among institutions or providers could affect the study’s outcomes and limit the applicability of findings to different settings or surgical procedures. Moreover, the study may not have delved deeply into the mechanisms by which chewing gum influences bowel function, thereby leaving a gap in terms of understanding the underlying reasons for its effectiveness.

## Conclusions

This study was intended as a positive step toward reducing issues related to prompt and early ileus prevention. Our findings reveal that sham feeding accelerates the recovery of gastrointestinal motility after CS because it reduces the amount of time needed for bowel sounds to appear and for stools and flatus to pass, leading to shorter hospital stays, less discomfort, and an earlier return to normal activities. Gum chewing has been associated with faster gastrointestinal motility recovery in patients with delayed gut motility after CS, and hence we recommend it as a standard postoperative strategy to encourage gastric motility in women who have had CS.
